# Combined effects of hyperglycemic conditions and HIV-1 Nef: a potential model for induced HIV neuropathogenesis

**DOI:** 10.1186/1743-422X-6-183

**Published:** 2009-10-30

**Authors:** Edward A Acheampong, Cassandra Roschel, Muhammad Mukhtar, Alagarsamy Srinivasan, Mohammad Rafi, Roger J Pomerantz, Zahida Parveen

**Affiliations:** 1 The Dorrance H. Hamilton Laboratories, Division of Infectious Diseases and Environmental Medicine, PA 19107, USA; 2 Bioscience Technologies - Biotechnology, Thomas Jefferson University, Philadelphia, PA 19107, USA; 3 Department of Biochemistry, Pir Mehr Ali Shah Arid Agriculture University Rawalpindi, 46300 Pakistan; 4 NanoBio Diagnostics, West Chester, PA 19382, USA; 5 Department of Neurology, Jefferson Medical College, Thomas Jefferson University, Philadelphia, PA 19107, USA; 6 Tibotec Inc. 1020 Stony Hill Road, Suite 300, Yardley, PA 19067, USA

## Abstract

Hyperglycemic conditions associated with diabetes mellitus (DM) or with the use of antiretroviral therapy may increase the risk of central nervous system (CNS) disorders in HIV-1 infected patients. In support of this hypothesis, we investigated the combined effects of hyperglycemic conditions and HIV-1 accessory protein Nef on the CNS using both *in vitro *and *in vivo *models. Astrocytes, the most abundant glial cell type required for normal synaptic transmission and other functions were selected for our *in vitro *study. The results show that *in vitro *hyperglycemic conditions enhance the expression of proinflammatory cytokines including caspase-3, complement factor 3 (C3), and the production of total nitrate and 8-iso-PGF2 α as reactive oxygen species (ROS) in human astrocytes leading to cell death in a dose-dependent manner. Delivery of purified recombinant HIV-1 Nef protein, or Nef expressed via HIV-1-based vectors in astrocytes showed similar results. The expression of Nef protein delivered via HIV-1 vectors in combination with hyperglycemia further augmented the production of ROS, C3, activation of caspase-3, modulation of filamentous protein (F-protein), depolarization of the mitochondria, and loss of astrocytes. To further verify the effects of hyperglycemia and HIV-1 Nef protein on CNS individually or in combination, *in vivo *studies were performed in streptozotocin (STZ) induced diabetic mice, by injecting HIV-1 Nef expressing viral particles into the sub-cortical region of the brain. Our *in vivo *results were similar to *in vitro *findings indicating an enhanced production of caspases-3, ROS (lipid oxidation and total nitrate), and C3 in the brain tissues of these animals. Interestingly, the delivery of HIV-1 Nef protein alone caused similar damage to CNS as augmented by hyperglycemia conditions. Taken together, the data suggests that HIV-1 infected individuals with hyperglycemia could potentially be at a higher risk of developing CNS related complications.

## Introduction

Antiretroviral therapy has been linked to insulin resistance and dyslipidemia in HIV infected individuals under treatment [1-4]. Since glucose is a major nutrient utilized by the brain[5], diabetes or HAART-associated hyperglycemic conditions may become a potential risk factor in the brain [6-8], and could lead to a series of devastating clinical conditions in the CNS of HIV-1 infected individuals[9]. Several studies have described hyperglycemia-induced neuronal and astrocytic glial cell death leading to various neurological disorders in diabetic patients[7,10,11]. However, limited information is available regarding the combined effects of hyperglycemia and HIV-1 infection on the CNS. Astrocytes play a critical role in the provision of nutrients and strength to the CNS via the foot processes protecting the blood brain barrier [12]. In this study, we selected astrocytes as target cells to evaluate the cumulative toxic effects of hyperglycemia and HIV-1 Nef protein. Previous studies have shown that hyperglycemia increases the production of proinflammatory cytokines, oxidative reactive species and activation of CD4+ and CD8 T lymphocytes in the peripheral blood system [13]. Of the proteins encoded by HIV-1, Env, Vpr, Vif, Tat, and Nef are known to exhibit cytopathic effects [14-16]. Specifically, the data from previous studies suggest a potentially important role of Nef in cellular dysfunctions and its contribution to the development of the neuropathology associated with AIDS. HIV-1 Nef expression has been shown to be essential in maintaining high replication level of the virus and promoting the development of AIDS in SIV-infected monkeys[17]. Skowronski and others have shown that the expression of Nef in transgenic mice is associated with the development of a severe AIDS like disease [18,19]. Nef and gp120 have been detected in the CSF of HIV-1 infected individuals and are known to be involved in the induction of complement factor C3 [9,20,21]. HIV-1 infection, thus affects the cellular processes in the brain by activating signaling pathways and the production of cytokines [22,23]. It has been reported that extracellular release of Nef protein could exert its effects on non-infected bystander cells in brain tissues of HIV-1 infected individuals and could be detected in distant brain regions [14,17]. HIV-1 proteins also cause an increase in systemic oxidative/nitrosative stress, by enhancing the deleterious effects of secondary infections [9]. The molecular mechanism involved in HIV-1 associated neuropathogenesis is not completely understood due to the inaccessibility of the brain parenchyma during the course of AIDS. Hence, limited information is available regarding the contributions of Nef alone and or in combination with hyperglycemic conditions to the pathogenesis of the CNS in the context of HIV-1 infection. The focus of this study was to evaluate the cytopathic effects of hyperglycemic conditions in the presence of HIV-1 Nef delivered either through HIV-1-based vector systems (intracellular) or in the form of recombinant protein (extracellular) in human astrocytes (*in vitro) *and STZ induced diabetic mice used as an *in vivo *model[24]. The delivery of Nef protein via viral injection into the STZ induced diabetic mice brain increased oxidative reactions as well as the production of inflammatory cytokines, complement factor C3, and depolarization of mitochondria. Induction of *in vitro *and *in vivo *hyperglycemia alone induced similar cytopathic effects in astrocytes and in diabetes induced mice. Further, the data involving astrocytes suggests that the presence of extracellular Nef protein further increased the risk of toxicity and cell death in a dose-dependent manner under hyperglycemic conditions.

## Materials and methods

### Cell Culture

Primary cultures of human fetal brain astrocytes and astrocytes medium were purchased from Cambrex, Inc (Walkersville, MD) and Sciencell (San Diego, CA). The cells were maintained in astrocyte media (AM) in a water-jacketed incubator at 37°C, with 5% CO_2 _in a humid environment. The cells were passaged at a confluence of 80-85%. The human glioblastoma/astrocytoma cell line U87-MG, and human kidney cell line 293-T were obtained from American Type Culture Collection (ATCC) and cultured in Dubelcco's Modified Eagle's Medium (DMEM) supplemented with 10% fetal bovine serum (Sigma Aldrich, St. Louis, MO), penicillin-streptomycin (100 U/ml and 100 μg/ml, respectively), and 2 mM L-glutamate (Mediatech Corp, MD).

### Generation of Nef expressing viral particles andtransduction

HIV-1 Nef expressing recombinant viral particles were generated by triple transfection of plasmids using Calcium phosphate transfection kit (Promega Corp, Madison, WI) following the manufacturer's protocol. Briefly, 293 T cells were seeded in 100 mm culture plates overnight. The cells were transfected with reagents of Mammalian Calcium Phosphate transfection kit (from Promega) in the presence of HIV-1 based vectors DNA; pHR'CMV Nef, pHR CMV delta 8.2, and pMD.G encoding VSV.G as an envelope protein. In addition, viral particles expressing HIV-1 Nef generated from spleen necrosis virus (SNV) packaging vector pZP^32^, transfer vector expressing HIV-1 Nef pZP^35^, and envelope vector VSV.G [14,25] were used as control. The supernatants from HIV-1 and SNV based viral vectors were harvested 3 days post transfection and frozen at -80°C. For some experiments both viruses were concentrated by ultracentrifugation at 25,000 rpm for one hour. The pellets were resuspended in 1% phosphate-buffered saline (PBS) containing 5% sucrose and stored at -80°C. The viral yield for HIV-1 was determined by p24 antigen enzyme-linked immunosorbent assay (ELISA) kit (Perkin Elmer, Boston, MA). Based on the quantification, equal amount of viral particles were used for the experiments. Culture supernatants without hyperglycemia and Nef were collected from the astrocytes and used as a source for mock treatment. Astrocytes were plated at 60% confluency over night before tranduction. Primary human astrocytes or U87-MG cells were distributed into 4-well chamber slides or plates at a cell density of 1.0 × 10^5 ^cells per well and allowed to stabilize in AM media for 24 hours prior to the addition of glucose media. After the stabilization period, the cells in each well were washed with pre-warmed 1× PBS. To mimic the *in vivo *hyperglycemia, glucose stock solutions were added to glucose-free media (contained 1.0 mM sodium pyruvate, 1% strep/pen, and 5% FBS) to achieve 10 mM, 15 mM, and 20 mM glucose concentrations. Of note, 10, 15, and 20 mM glucose represents the 180, 200, 350 mg glucose/dl blood in diabetic patients. The medium with 5.0 mM glucose was used as a control. The astrocytes were exposed to *in vitro *hyperglycemic conditions for 12 hours and washed with 1× PBS. The astrocytes were then transduced with viral supernatant mixed with 8 ug/ml polybrene for astrocytic cell line(U87-MG) and 4 ug/ml for primary astrocytes for 3 hours followed by washing to remove the virus and incubated with complete medium. Astrocytes were harvested and supernatants were collected after 48 hours for various analyses. Non- transduced astrocytes were used as a control.

### *In vitro *effects of hyperglycemia and recombinant Nef protein on human astrocytes

Individual and cummulative effects of hyperglycemia and recombinant Nef protein on primary human fetal astrocytes were evaluated by observing changes in the F-actin, a protein involved in mitochondrial and cellular integrity [26]. Astrocytes were seeded into 4-well chamber slides at a cell density of 1.0 × 10^5 ^cells per well and exposed to various hyperglycemic conditions for 12 hours, followed by extensive washing with 1× PBS. The cells were fixed with 4% paraformaldehyde for 10 minutes and washed several times with 1× PBS to remove the fixative. The astrocytes were then stained with BODIPY phallacidin (Invitrogen Corporation, Carsbad, CA) cytoskeleton staining dye following protocol suggested by the manufacturer, and observed under fluorescence-microscope.

The effect of recombinant Nef protein on mitochondria was studied by using Mitotracker dye (Invitrogen Corporation, Carsbad, CA). The dye stains mitochondria only upon depolarization. For this, astrocytes were seeded in chamber slides in AM medium and allowed to attach for 24 hours prior to Nef protein treatments. Recombinant Nef protein generated in our laboratory [14] was added at concentrations of 1 nM, 3 nM, and 25 nM in 500 μl of glucose free DMEM containing 1.0 mM sodium pyruvate, 1% strep/pen, and 5% FBS and incubated with astrocytes for 24 hours. The cells were washed with 1XPBS and stained live with 200 nM Mitotracker Red fluorochrome (Invitrogen Corp., Carlsbald, CA), to detect the depolarization of mitochondria.

### Induction of Diabetes

Mice with C57/BL6 genetic background were purchased from Jackson Laboratories to study the effects of hyperglycemic variations either alone or in combination with intracellularly expressed HIV-1 Nef protein *in vivo*[27]. To rule out the effect of other accessory proteins associated with HIV-1 virus, SNV vector virus encoding Nef was also included in the study. The exact physiological concentration of Nef is not clear in HIV-1 infected individuals. However extracellular recombinant Nef protein generated in our laboratory was added to astrocytes at concentrations mentioned in previous studies [28]. Diabetes was induced in 12 mice by a single subcutaneous injection of 40 mg/kg body weight streptozotocin (Sigma Aldrich Corp., St.Louis, MO) dissolved in freshly prepared 0.1 M citrate buffer pH 4.5. The blood glucose level was assessed by a glucometer using a drop of blood drawn at 1, 2, 4, 6, 8, 10, and 12 hours post injection. The mean elevated glucose level was 325 mg/dl after injection of STZ. Upon confirmation of induction of hyperglycemia in mice, 2-ul of concentrated viral particles (1 × 10^7^) generated through HIV-1 and SNV-based vectors systems were injected into the brain of mice via the cortex as described previously [29]. Age-matched non-diabetic and STZ treated (diabetes) mice injected with an equal volume of citrate buffer, served as controls. The animals were housed under pathogenic free conditions in Thomas Jefferson Animal Facility. Mice from the ages of 1-2 weeks of the same sex were used in the experiments. All procedures were conducted in accordance with federal guidelines using animal protocols approved by the Thomas Jefferson University Institutional Animal Care and Use Committee (IACUC). The mice were sacrificed eight weeks post-injection and their brains were detached, washed in cold 1× PBS and used for various analyses (25).

### Western Blot Analyses

Astrocytes exposed to various glucose solutions to induce hyperglycemia, or in combination with HIV-1 Nef, or transduced with Nef alone, as well as non-treated control astrocytes were washed and lysed in radio Immunoprecipitation assay (RIPA) buffer containing protease inhibitors. The protein concentrations were determined with the bicinchoninic acid protein assay kit (Pierce Biotechnologies, Rockford, IL). Approximately 25 μg of each protein preparation was resolved on 10% sodium dodecyl sulfate polyacrylamide gels (Bio-Rad) and transferred to polyvinylidene difluoride (PVDF) membranes (Amersham Biosciences, Piscataway, NJ) using electroblotting method. The membranes were washed in PBS containing 0.01% Tween 20 (Sigma-Aldrich, St. Louis, MO.). Non-specific proteins were blocked with PBS-based blocking buffer (Pierce Biotechnologies, Rockford, IL) and the membranes were probed with specific monoclonal antibodies against GFAP at a concentration of 1:1000, mouse anti-Caspase 3 antibody at a concentration of 1:1000 as primary antibodies and horseradish peroxidase labeled anti-mouse immunoglobulin G (heavy plus light chains) as secondary antibodies. The protein-antibody complexes were visualized by autoradiography of the membranes after incubating with the ECL blotting detection system (Pierce Biotechnologies, Rockford, IL) and subsequently exposing them to BioMax MS (Kodak, Rochester, N.Y.) film. HIV-1 Nef protein was detected in the transduced astrocytes by treating the blots with anti-HIV-1 Nef antibody (NIH AIDS Repository). For *in vivo *analysis of the expression of Nef, viral vector expressing Nef was identified from mice brain tissues using immunoprecipitation method. The Seize X Immunoprecipitation Kit (Pierce Biotechnologies, Rockford, IL) was used following the manufacturer's protocol. The purified Nef protein was then subjected to Western Blot analysis using the method described earlier.

### Enzyme Linked Immunosorbent Assays (ELISAs)

The production of nitric oxide and lipid oxidation reaction in the form of total nitrate and 8-iso- PGF-2α, respectively, were measured to determine the level of reactive oxidative species induced as a result of exposure to either hyperglycemic conditions alone or in combination with HIV-1 Nef protein or Nef alone. The U87-MG cells were exposed to various concentrations of glucose (10, 15, 20, 25 mM glucose solutions) followed by transduction with HIV-1 Nef expressing viral particles. Control astrocytes were cultured in normal medium. For *in vivo *studies, 10-day-old mice were injected with a single dose of STZ for induction of hyperglycemia followed by delivery of HIV-1 based Nef expressing virus via injection in the brain tissues. Non-diabetic mice, hyperglycemic mice, or HIV-1 Nef injected mice were used as controls. To rule out the influence of other accessory proteins of HIV-1 and for exclusive effect of Nef protein, mice were also injected with virus generated by SNV vector systems [27]. The cell/tissue lysates and supernatants from the treated cells as well as from brain tissues from the hyperglycemic and Nef treated mice were collected. Samples were analyzed for the presence of nitric oxide (NO), 8-isoprostaglandin-F2-α, or complement factor C3 using respective ELISA kits (Stressgen Biotechnologies, Victoria, BC, Canada) as well as the manufacturer's suggested protocol [30].

## Results

In this study, we utilized *in vitro *and *in vivo *models to evaluate the combined cytopathic effects of hyperglycemia and HIV-1 proteins on the CNS to mimic the conditions in individuals with diabetes or hyperglycemia associated with the use of highly active antiretroviral therapy (HAART). For *in vitro *studies, U87-MG/primary astrocytes were exposed to various hyperglycemic conditions by adding the appropriate amount of glucose in medium [31] and tranduced with HIV-1 Nef expressing virus. For the *in vivo *studies, diabetes was induced in mice with STZ and recombinant viral particles expressing HIV-1 Nef were injected in both STZ induced diabetic and normal mice brains. The combined cytopathic effects of hyperglycemia and HIV-1 Nef protein on the CNS were determined by evaluating the expression of complement factor 3, production of oxidative species (ROS), caspase activity, changes in F-actin protein, and the depolarization of mitochondria.

### Effect of Hyperglycemia and HIV-1 Nef on Complement Factor 3(C3)

**Figure 1:**
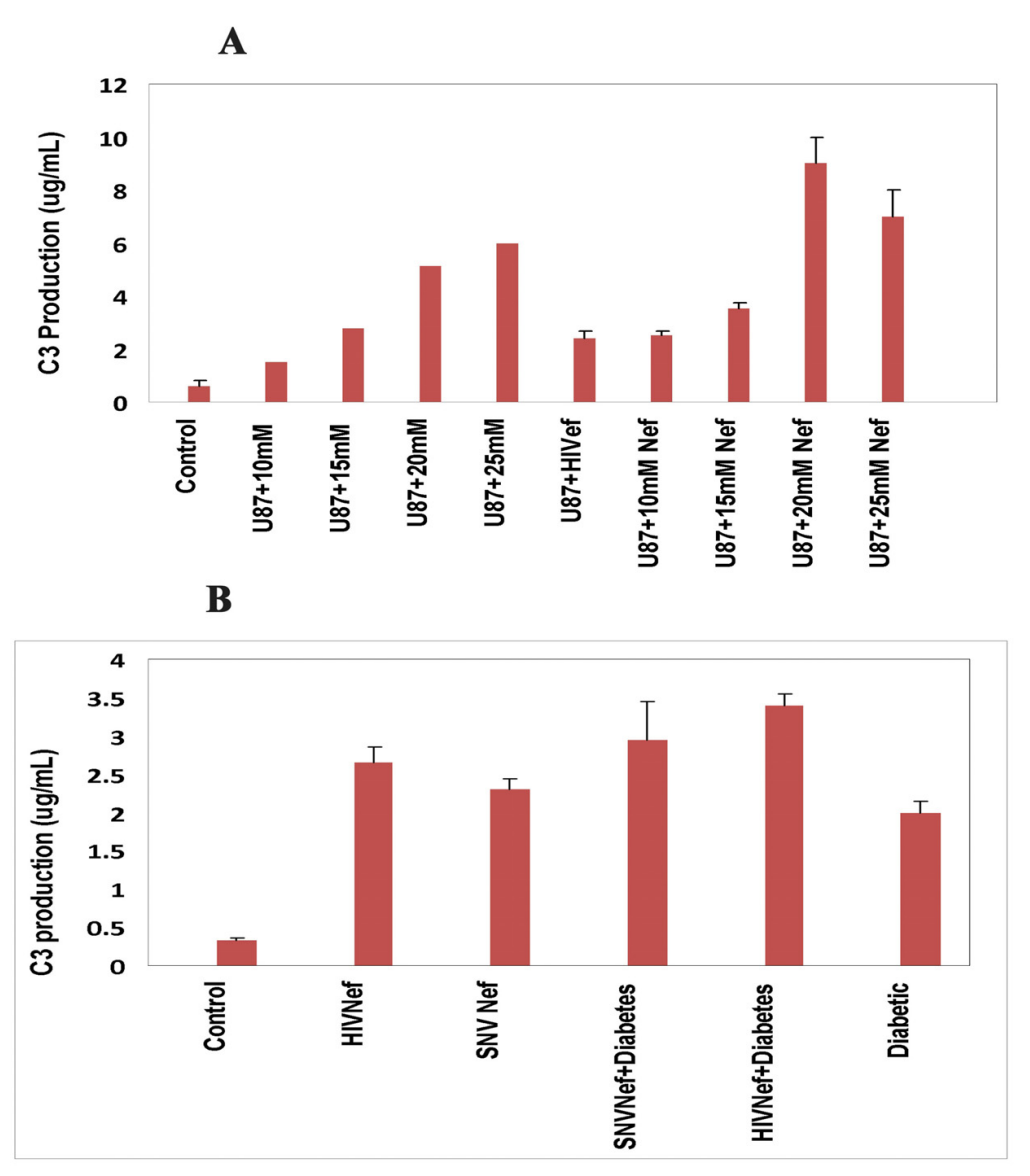
**Hyperglycemic conditions and HIV-1 Nef significantly enhance the production of complement factor C3 *in vitro *and *in vivo***. (A) To mimic hyperglycemic conditions close to the blood glucose levels of 180, 270 and 360 mg/dl, U87-MG human astroglioma cells were cultured with 10,15, 20 and 25 mM glucose containing medium for 12 hours. Astrocytes with 5 mM glucose treatment were used as control. The cells were washed and transduced with HIV-1 Nef expressing virus. 48 hours later, the astrocytes and cellular supernatants were collected and subjected to ELISA using manufacturer's protocol to quantify the complement factor 3 (Assay Designs, Ann Arbor, MI) (B). Hyperglycemic conditions and expression of HIV-1 Nef significantly enhanced the production of complement factor 3 in mice brain. Diabetes was induced in C57/BL6 mice by a subcutaneous injection of a single dose of 40 mg/kg body weight streptozotocin (Sigma Chemicals, St.Louis, MO), which has been freshly dissolved in 0.1 mol/L citrate buffer at pH of 4.5. Upon confirmation of diabetes induction (325-425 mg/dl glucose) by a glucometer in these mice, 1 × 10^7 ^viral particles generated through HIV-1 based vectors or SNV-based vectors were injected into the mice brain via the mid ventricle, cortex, or the cerebellum as described previously. Age-matched non-diabetic mice injected with an equal volume of citrate buffer were served as control. After eight weeks the mice were sacrificed and the brain and other organs were harvested. ELISA was performed on brain tissue extracts to determine the release of C3 into the brain. The results are the mean values for triplicate samples ± standard errors of the means. The data presented are averages of three independent experiments.

The cerebral complement system has been known as a contributor to AIDS-associated neurological disorders. To evaluate the inflammatory response during hyperglycemia and/or HIV- infection in the CNS, complement factor 3 was used as an indirect measure of immune response [20,21]. Our results indicate that the exposure of astrocytes to 10, 15, 20 and 25 mM glucose increased the expression of C3 (2.0, 4.0, 10 and 10.7 fold) in a dose-dependent fashion respectively. Expression of Nef via HIV-1 vectors in astrocytes exposed to 10, 15, 20 and 25 mM glucose resulted in an increase of more than 4.0, 6.0, 16 and 12 fold respectively in the production of C3 (Figure 1A). Exposure of astrocytes to HIV-1 Nef alone also enhanced the production of C3 to more than 4 fold, suggesting that HIV-1 Nef itself is capable of inducing immune response[20]. The effect of hyperglycemia on C3 production was also studied *in vivo *using STZ-induced diabetic mouse model. Our results from diabetic mice were similar to the results obtained from the *in vitro *study in astrocytes. We observed more than 6-fold increase in the production of C3 in diabetic mice brain as compared to the normal mice. The expression of Nef particles delivered via injection into normal mice brain resulted in 8-fold increase in C3, while the expression of Nef in diabetic mice resulted in more than 10-fold increase in C3 production (Figure 1B) as compared to normal mice used as control. Delivery of HIV-1 Nef via SNV vectors into mice brain also showed similar increase in C3, suggesting the exclusive effect of Nef in enhancing the C3 production. These results indicate that *in vitro *hyperglycemia or *in vivo *diabetic conditions increase the immune response in the form of complement factor 3 production in CNS, whereas the expression of Nef under normal glycemia or in combination with hyperglycemia further enhanced the production of C3 as a consequence of severe immune reaction [12].

### Detection of Reactive Oxygen Species (ROS)

#### Effect of Hyperglycemia and HIV-1 Nef on Nitric Oxide Production

**Figure 2:**
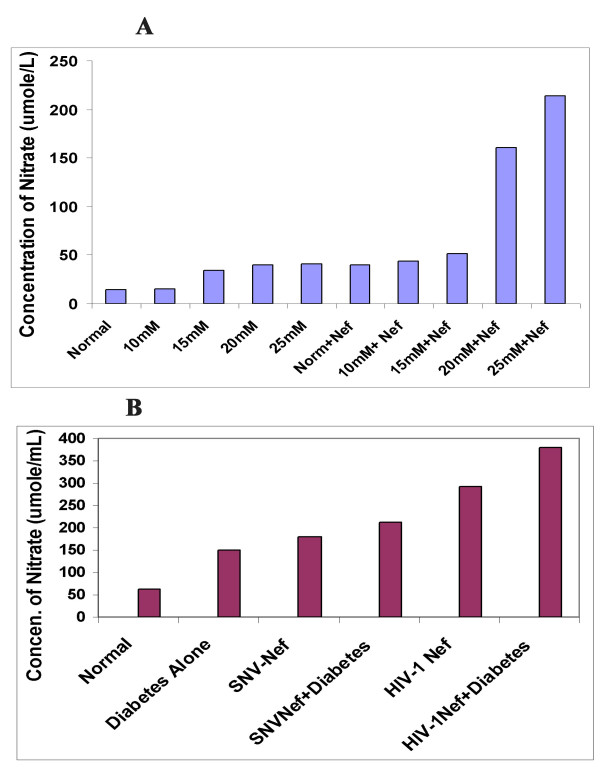
**Hyperglycemia and HIV-1 Nef significantly enhanced the production of nitric oxide in the CNS *in vitro *and *in vivo***. (A) Hyperglycemia and HIV-1 Nef enhanced the production of nitric oxide in human primary astrocytes (*in vitro*) in dose dependent fashion. Primary human astrocytes were cultured and exposed to glucose solutions for12 hours as indicated earlier. Astrocytes with 5 mM glucose containing medium were used as control. After exposure to glucose, the astrocytes were transduced with HIV-1 Nef expressing virus. 48 hours later, the Nef-transduced astrocytes and cellular supernatants were collected and oxidative stress was determined by measuring the release of nitric acid in the astrocytes and in the supernatant with an ELISA kit (Stressgen, Victoria, BC, Canada). (B) Hyperglycemia and HIV-1 Nef significantly enhanced the production of nitric acid in mice brain: 1 × 10^7 ^viral particles generated through HIV-1 vectors or SNV vectors were injected into the brain of diabetes-induced mice via the cortex as described previously (Parveen et al 2003). Age-matched non-diabetic mice injected with an equal volume of citrate buffer served as control. After 8 weeks, the mice were sacrificed and the brain tissue lysates were subjected to ELISA to determine the release of total nitrate in the brain. The results depicted in this figure clearly indicate that hyperglycemia and Nef, either alone or in combination enhance oxidation reaction by increasing the release of total nitrates in CNS. The results are mean values of duplicate samples.

The ability of hyperglycemia to induce reactive oxygen species (ROS) thereby enhancing the production of nitric oxide (total nitrate) and lipid peroxidation in the form of 8-iso-prostaglandin F2 alpha (8-iso-PGF2 alpha) are well documented, and have been previously used as biological markers to detect the oxidative stress levels [14,32,33]. In this study, we investigated the production of reactive oxygen species by determining the level of total nitrates produced due to *in vitro *hyperglycemic conditions or due to the expression of HIV-1 Nef protein in astrocytes or combination of both, as well as *in vivo *in diabetic mice. Figure 2A shows that hyperglycemia doubled the concentration of total nitrate in astrocytes upon exposure to 15, 20 and 25 mM glucose respectively, with the exception of 10 mM glucose showing similar nitrate level as observed in astrocytes cultured under normal glycemic conditions. U87-MG astrocytes transduced with Nef expressing virus alone showed more than 2-fold increase in total nitrate. The combination of hyperglycemia with Nef expressing virus in astrocytes resulted in a dose- dependent increase in total nitrate. Astrocytes exposed to 10, 15, 20 and 25 mM glucose, and transduced with HIV-1 Nef expressing virus, increased the total nitrate from 3, 3.5, 11 and 15 fold respectively. These results suggest that hyperglycemia and Nef alone or in combination induce oxidative stress in the CNS in dose-dependent fashion.

To confirm our *in vitro *results *in vivo*, a total of 24 mice were used in the study. Diabetes was induced in 12 well-characterized C57/BL6 genetic background mice by injecting a single dose of STZ. HIV-1 Nef expressing viral particles or SNV based viral particles expressing Nef were injected into the cortex region of eight mice brains while the remaining four diabetic mice continued to grow for eight weeks. In addition, six normal mice were injected with HIV-1 Nef and SNV Nef expressing viral particles into the cortex region of the brain. Four untreated normal mice were used as controls. Eight weeks later, the mice were sacrificed to analyze the effects of hyperglycemia alone or in combination with HIV-1 Nef protein expressed via HIV-1 based vectors on the CNS. The results illustrated in Figure 2B show similarities in the *in vitro *and *in vivo *increase in total nitrate. The hyperglycemic conditions also increased (2-fold) the total nitrate in diabetic mice brain. Delivery of HIV-1 based Nef expressing virus into the brain of the diabetic mice further enhanced the total nitrate production (more than 6-fold), in comparison to non-diabetic control mice. HIV-1 Nef expressing particles delivered into normal mice brain showed more than 4-fold increase in the production of total nitrate as compared to the normal mice. Overall, our *in vivo *results are in agreement with those obtained through *in vitro *studies in astrocytes. Further, to rule out the impact of other HIV-1 accessory proteins, Nef expressing recombinant retroviral particles were generated using spleen necrosis virus vectors and injected into the cortex of mice brain and the results are depicted in Figure 2B. These results are close to those obtained when viral particles expressing HIV-1Nef were injected into the brain of mice. Delivery of SNV-based Nef expressing virus alone increased more than 2-fold of total nitrate in normal mice brain, while diabetic mice showed 3-fold increase in total nitrate. These results are suggestive of an exclusive effect of Nef protein on astrocytes.

#### Effect of hyperglycemia and HIV-1 Nef on 8-iso-PGF2 α, production

**Figure 3:**
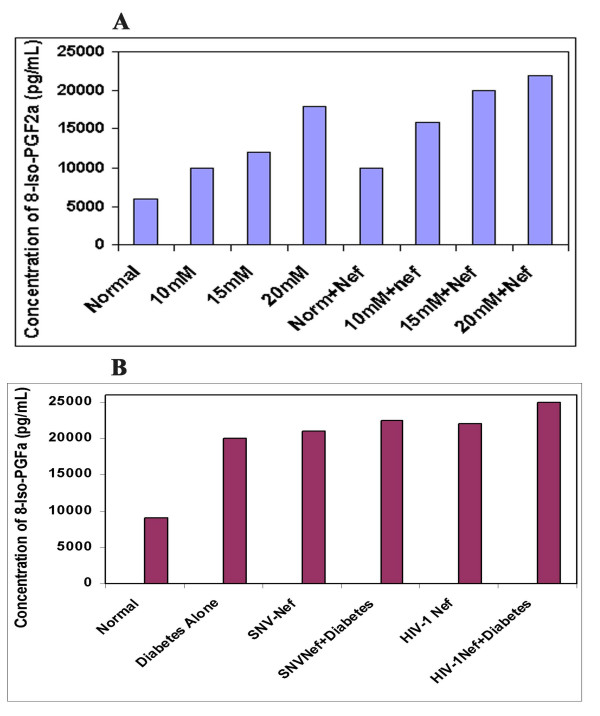
**Hyperglycemia and HIV-1 Nef significantly enhanced lipid oxidation in the CNS *in vitro *and *in vivo***. (A). Primary human astrocytes were cultured with 10,15, 20 and 25 mM glucose containing medium for 12 hours. The cells were then transduced HIV-1 nef expressing viral particles. 48 hours later, the Nef transduced astrocytes and cellular supernatants were collected and the lipid oxidation was determined by measuring the production of 8-isoprostaglandin-F2- α using ELISA kit (Stressgen, Victoria, BC, Canada). Astrocytes without any additional glucose (5 mM) treatment were used as control. Our results indicate that hyperglycemia increased the production of 8-isoprostaglandin-F2- α in dose dependent manner and Nef alone also showed a 3-fold increase in 8-isoprostaglandin-F2- α. (B) Hyperglycemia and HIV-1 Nef significantly enhanced the production of 8-isoprostaglandin-F2- α in the brain of mice: 1 × 10^7 ^viral particles generated through HIV-1 vectors or SNV vectors were injected into the brain of diabetes-induced mice via the cortex as described before. Age-matched non-diabetic mice injected with an equal volume of citrate buffer served as control. After 8 weeks the mice were sacrificed and lysates from the brain tissues were subjected to ELISA to determine the release of 8-isoprostaglandin-F2- α. The results depicted in this figure indicate that hyperglycemia enhanced the production of 8iso-F2- α in a dose-dependent manner and HIV-1 Nef either alone or in combination with hyperglycemia also enhanced the release of 8-isoprostaglandin-F2- α in CNS causing oxidative stress. The results are the mean value of triplicate samples.

The effect of hyperglycemia and HIV-1 Nef on lipid oxidation in astrocytes was determined by measuring the 8-isoprostaglandin (8-iso-PGF2 alpha) using ELISA techniques. Astrocytes treated with various hyperglycemic conditions were either analyzed within seventy-two hours post treatment or were transduced with Nef expressing viral particles. The supernatants were collected and analyzed for the production of 8-iso-PGF2-α. Similarly, diabetes-induced mice were either left untreated or injected with HIV-1 Nef expressing virus. Eight weeks post-injection, the mice were sacrificed and the brains were removed to analyze the cortex region of the brain for the production of 8-iso-PGF2 **α**. The results of both experiments are presented in Figure 3A and 3B. Fig. 3A, depicts the effect of hyperglycemia alone or in combination with HIV-1 Nef protein, in astrocytes indicating an enhanced production of 8-iso-PGF2 **α **in a dose-dependent manner. Hyperglycemic conditions alone increased the release of 8-iso-PGF2 **α**, ranging from 2 to 3-fold in astrocytes exposed to 10,15 20 and 25 mM glucose. The combination of hyperglycemia and HIV-1Nef both resulted in more than 3 to 4-fold increase in lipid peroxidation reaction. The astrocytes treated with 25 mM glucose and transduced with Nef were indicating increase in cell death. Fig 3B, depicts the effect of diabetes and Nef on the production of 8-iso-PGF2 **α**. Our results indicate that induction of hyperglycemia in mice brain increased the production and release of 8-iso-PGF2 **α**, and delivery of HIV-1 based Nef expressing particles further enhanced it (2-fold further increase), as compared to the control mice. Furthermore, the use of SNV-based Nef expressing virus as a means of ruling out the possible added effects of other HIV-1 proteins and also to demonstrate the exclusive effect of HIV-1 Nef protein, produced similar results (Figure 3B). The expression of Nef alone in the brain of mice showed a similar increase (8-fold) in 8-iso-PGF2 **α **as compared to the control mice.

#### Effect of Hyperglycemia and HIV-1 Nef on Cytoskeleton and Mitochondria

**Figure 4:**
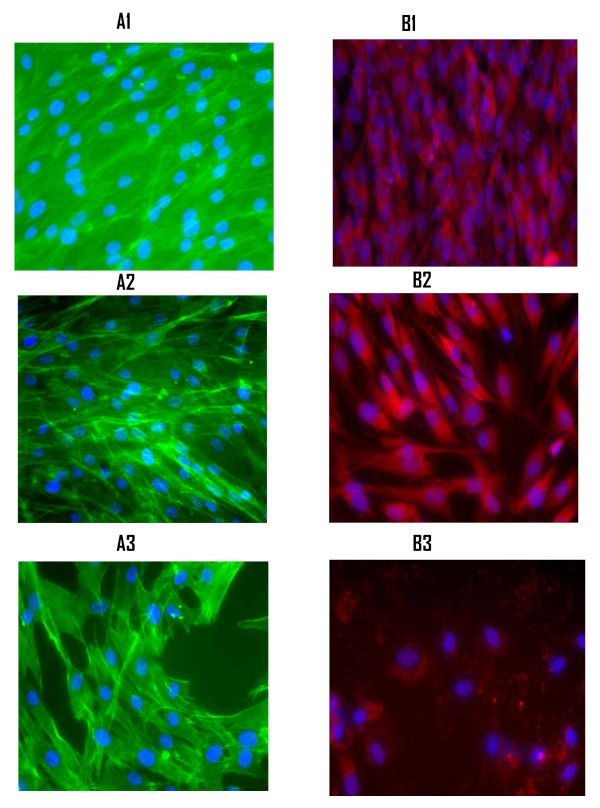
**Effect of Hyperglycemia and HIV-1 Nef on Cytoskeleton and Mitochondria of astrocytes**. Primary human astrocytes were cultured and exposed to various hyperglycemic conditions for 12 hours as mentioned before followed by washing with 1× PBS. The cells were then fixed with 4% paraformaldehyde for 10 minutes, and washed again with 1× PBS to remove the fixative. The effect of hyperglycemia on the cytoskeleton network (F-actin protein) was observed by staining the cells with phallacidin using protocol provided by the manufacturer, and examined under the fluorescent microscope. Panel A1-A3: A1. Astrocytes grown in normal medium, which served as control was stained with BODIPY phallacidin illustrate the normal cytoskeleton network. A2: Astrocytes treated with 15 mM glucose illustrates loose F-actin network and increased intracellular space indicating the loss of astrocytes. A3. Astrocytes treated with 25 mM glucose indicate significant changes in the cytoskeleton. The F-actin network was expanded and the intracellular space in between the astrocytes was further increased indicating cell death under higher glycemic conditions. **B1**. Normal astrocytes stained with MitoTracker Red to observe the effect of extracellular HIV-1 Nef recombinant protein on mitochondria. A2. 3 nM Nef protein solution was added into the medium with astrocytes and stained with MitoTracker. A3. Highly polarized mitochondria of primary astrocytes upon exposure to 25 nM of recombinant Nef protein, suggesting that free Nef protein could cause mitochondrial depolarization and ultimately cell death.

Previous studies have shown that an increase in F-actin protein dynamics correlates with increase in ROS levels in astrocytes, which has been involved in depolarization of mitochondria[26]. The impact of hyperglycemia and HIV-1 Nef on F-acting protein was investigated using fluorescence actin-labeling reagent Bodipy phallacidin [34]. Whereas mitochondrial depolarization was detected with Mitotracker Red fluorochrome [35] dye. The impact of hyperglycemia on the network of F-actin protein of astrocytes exposed to various glucose solutions was studied 72 hours after 12 hours exposure to glucose. The astrocytes were washed and stained with phallacidin dye following observation under microscope. Our results show a dense network of cytoskeleton and F-actin protein in astrocytes under normal glycemia (Figure 4, panel A1). Exposure of astrocytes to various concentrations of glucose ranging from 15 mM to 20 mM enhanced the visibility of F-actin protein with significant changes in the cytoskeletal structure as depicted in Figure 4 panels A2 and A3. The actin-network in astrocytes exposed to 15 mM glucose was very visible with expanded cell structure. Exposure to 20 mM glucose further enhanced the visibility with a higher degree of disorganization of actin-network cell expansion, and increase in intracellular space indicating loss of astrocytes (Figure 4, panel A3).

The mitochondrial depolarization was determined by MitoTracker Red fluorochrome detection method [35,36]. Figure 4 panel B2-3, depict an increase in the depolarization of mitochondria in a dose-dependent manner with the addition of 3 nM or 25 nM/ml of recombinant Nef protein. The addition of 3 nM/ml Nef protein caused the depletion of astrocytes, whereas addition of 25 ng Nef completely damaged the astrocytes layer (Figure 4 panel B3) suggesting that intracellular accumulation of Nef (due to an increase in HIV-1 replication) in astrocytes could trigger apoptosis and a non-reversible damage of the mitochondria [37].

#### Effect of Hyperglycemia and Nef on caspases

**Figure 5:**
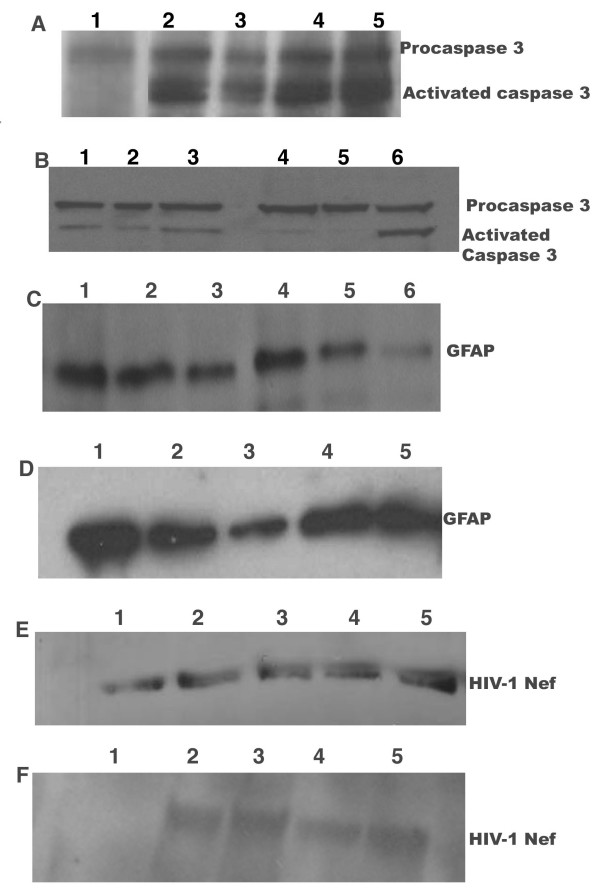
**Effect of hyperglycemia and HIV-1 Nef on caspases and GFAP protein**. For *in vivo *studies, 10 day old and STZ induced diabetic mouse pups were injected with 1 × 10^7 ^HIV-Nef infectious particles generated from HIV-1 or SNV based vectors systems. The pups were sacrificed 8 week after the injections. The brain tissue sections from cortex were removed and cellular protein lysates were prepared and loaded (25 μg/lane) onto a sodium dodecyl sulfate-(SDS) gel and electrophoresed, followed by a transfer onto a nitrocellulose membrane. The blots were then probed with antibody specific for whole and activated caspase-3. Panel A. Lane 1, Normal mice brain tissue protein serving as control, lane 2. Non-diabetic mice brain injected with HIV-1 Nef particles. Lane 3, diabetic mice brain tissues, lane 4, diabetic mice brain with SNV based Nef expressing virus, lane 5, diabetic mice brain with HIV-1 Nef expressing virus. Panel B. Astrocytes (U87-MG) were cultured under various glycemic conditions and transduced with HIV-1 Nef expressing viral particles. Forty-eight hours later, cells were lysed and the lysates (25 μg/lane) were loaded onto a SDS gel and electrophoresed, followed by a transfer onto a nitrocellulose membrane. The blots were then probed with antibody specific for whole and activated caspase-3. Lanes-1, expression of procaspase-3 in normal astrocytes transduced with HIV-1 Nef particles, 2-astrocytes treated with 10 mM glucose and HIV-1 Nef virus, 3-astrocytes treated with 15 mM glucose and HIV-1 Nef virus, 4-astrocytes treated with 18 mM glucose, 5-non treated normal astrocytes, 6-astroctes treated with 18 mM glucose and HIV-1 Nef virus. Panel C - Primary human astrocytes exposed to various hyperglycemic conditions and transduced with HIV-1 Nef expressing virus. The cells lysates are probe with GFAP antibody. Lanes: 1-non-treated normal astrocytes, 2-astrocytes treated with 18 mM glucose, 3- normal astrocytes transduced with HIV-1 Nef virus, 4- 10 mM glucose treated astrocytes, 5- astrocytes treated with 15 mM glucose and transduced with HIV-1 Nef virus, 6-astrocytes treated with 18 mM glucose and HIV-1 Nef. Panel D - Brain tissue lysates of diabetic or non-diabetic mice with HIV-1 Nef virus delivered into various regions of the brain. The tissues lysates were probed with antibody against GFAP. Lanes: 1-normal mice brain tissue, 2- diabetic mice brain tissue, 3-non-diabetic mice brain exposed to HIV-1 Nef virus, 4-diabetic mice with HIV-1 Nef virus, 5-diabetic mice with HIV-1Nef virus generated from SNV vectors. Panel E: Hyperglycemic treated and HIV-1 Nef-transduced astrocytic cell lysate probed with antibody specific against HIV-1 Nef protein. Lanes: 1-normal astrocytes, 2- astrocytes transduced with HIV-1 Nef virus, 3- astrocytes treated with 10 mM glucose and transduced with HIV-1 Nef virus, 4- astrocytes treated with 15 mM glucose and transduced with HIV-1 Nef virus, 5- astrocytes treated with 18 mM glucose and transduced with HIV-1 Nef virus, 6- astrocytes treated with 18 mM glucose and transduced with SNV Nef virus. Panel F: Brain tissue lysates of diabetic and non-diabetic mice injected with HIV-1 Nef virus and probed with Nef specific antibody. Lanes: 1- normal mice brain tissues, 2- mice injected with HIV-1 Nef virus, 3- diabetic mice injected with HIV-1 Nef virus, 4- diabetic mice injected with SNV based Nef virus, 5-normal mice injected with SNV-based HIV-1 Nef virus.

To determine whether HIV-1 Nef and hyperglycemic conditions induced apoptosis, intracellular activity of caspase -3 was analyzed in primary astrocytes exposed to HIV-1 Nef particles, via Western blot and the results are depicted in Figure 5 panel A and B. The figure illustrates the impact of hyperglycemia and Nef on mice brain (*in vivo*) and *in vitro *on U87-MG astrocytes respectively. Panel A, lane 1 represents the pro-caspase 3 in normal mice brain while lane 2 represents the activated caspase-3 as a result of HIV-1 Nef expressing viral particles. Lane 3 also depicts the activation of caspase -3 by hyperglycemia. To ensure that the apoptosis observed is the exclusive effect of HIV-1 Nef protein, we subjected the brain lysates from diabetic mice injected with SNV- based Nef particles to western blot analyses and compared the results with brain lysates of mice injected with HIV-1 Nef expressing particles (Figure 5 panel A lane 4 and lane 5). These results suggest that hyperglycemia and Nef have an additive effect on caspase-3 activity, which could induce apoptosis. In panel B, the *in vitro *results of hyperglycemic treated astyrocytes transduced with Nef exhibited dose-dependent activation of caspase-3 as depicted in Figure 5 panel B (lanes 2, 3 and 6), suggesting the apoptotic potential of hyperglycemic conditions which were dramatically augmented and synergized by Nef (Figure 5 panel B lanes 2, 3 and 6). We also observed that the expression of Nef alone triggers the activation of caspase -3 as illustrated in figure 5 panel B and lane 1. Similar observations were made in our *in vivo *studies as well. The apoptotic effect of hyperglycemia and HIV-1 Nef on astrocytes and on CNS was also determined by quantifying the glial fibril acidic protein (GFAP) using GFAP specific antibody. The western blot analyses of astrocytes and mice brain exposed to hyperglycemia and/or Nef are shown in figure 5 panels C and D respectively. These results indicate that astrocytes exposed to hyperglycemia have reduced GFAP expression as shown in panel C lane 2 compared to normal astrocytes in Figure 5 panel C and lane 1. The results also indicate that Nef alone is capable of down-modulating the expression of GFAP to a great extent in astrocytes than the hyperglycemia alone (Figure 5, panel C lane 3). Astrocytes exposed to various glucose solutions and transduced with HIV-1 Nef showed a dose-dependent decrease in GFAP protein expression (Figure 5 panel C lanes 4, 5 and 6) suggesting that hyperglycemic variations and Nef combination may synergistically and adversely affect the expression of GFAP in astrocytes. The GFAP expression in STZ treated mice brain with and without Nef expression was also evaluated and the results are presented in Figure 5 panel D. These *in vivo *results are in agreement with our *in vitro *results as evident in lane 1, 2, and 3 illustrating the expression of GFAP in normal mice brain, diabetic mice and mice brain injected with Nef expressing particles (Figure 5 Panel D, lanes 4 and 5). It is evident from our results that HIV-1 Nef is more efficient in down-modulating the expression of GFAP than hyperglycemic conditions. The data presented here also suggest that even low expression of HIV-1 Nef could affect astrocytes by reducing the GFAP expression [38]. The expression of Nef was also detected in astrocytes and in mice brain delivered via SNV based vectors, as shown in Figure 5 Panel E and F. All these results shown here are representative of at least three independent experiments and repeated several times.

## Discussion

The use of highly active antiretroviral therapy (HAART) has reduced the mortality and morbidity rates in HIV-1 infected individuals [39]. However, many disorders related to glucose metabolism and fat redistribution are becoming prevalent in HAART receiving patients [1-4,40,41]. Diabetes is an increasingly common disorder and causes a variety of central nervous system (CNS) complications including cognitive dysfunctions [6,7,10,32,42]. Glucose is one of the major nutrients utilized by the brain. Hyperglycemia/diabetes may allow the entry of immune cells into the CNS through impaired BBB, causing a series of devastating clinical conditions in the central nervous system (CNS) [6,8,11,32].

We therefore investigated the pathological state of CNS in association with hyperglycemia and HIV-1 Nef protein that has been implicated in AIDS neuropathogenesis by acting as a mediator to recruit leukocytes that may serve as vehicles of the virus and perpetrators for disease through the production of neurotoxins [43,44]. The *in vitro *studies were performed in primary human astrocytes and astrocytes cell line(U87-MG human glioma cell line). Astrocytes are highly abundant in the brain and play a vital role by providing the metabolic and protective support to neurons and to the blood brain barrier (BBB)[45]. Our results indicate that HIV-1 Nef and hyperglycemia, alone or together, induce elevated expression of C3 in astrocytes as well as in diabetes induced mice brain. The normal synthesis of C3, an antimicrobial defense mechanism in the brain, is usually low and the observed increase in its production after exposure to Nef or hyperglycemia alone or in combination suggests a very high immune response by astrocytes and by brain tissues[20,46].

In addition to the increased production of C3, we also have identified nitric oxide (NO) as a source of cellular oxidative stress induced in both astrocytes and brain tissues isolated from diabetes-induced mice. Under increased hyperglycemia, we observed increased expression of total nitrate in astrocytes in a dose-dependent manner compared to the control non-glucose treated astrocytes. Similarly, our *in vivo *diabetes-induced mice model also showed increase in nitrate as compared to that of normal mice. Furthermore, astrocytes exposed to hyperglycemic conditions particularly exposure to 20 and 25 mM glucose with HIV-1 Nef virus showed a synergistic increase in nitrate production in comparison to the control astrocytes. Similar results were obtained when HIV-1 Nef expressing virus was injected into the brains of diabetic mice and compared to non-diabetic mice injected with HIV-1 Nef virus (Figure 2B). Non-glucose treated astrocytes transduced with Nef virus also showed an increase in total nitrate, however, the level of production was relatively lower than that observed in astrocytes with hyperglycemia. These results are fully consistent with the results of other studies, which have shown that hyperglycemic conditions may contribute to CNS malformation via oxidative stress[33,47].

HIV-1 proteins have been shown to be involved in exacerbating oxidative and nitrosative stress [48-51], and our results also demonstrate that HIV-1 Nef increases oxidative stress both *in vivo *and *in vitro *models. Indeed, the development of HIV-1 associated dementia has been directly attributed to HIV-1-induced oxidative stress and the accompanying overproduction of several toxic factors, including prostaglandins, CD95 ligand, and free radicals [52-58].

We are reporting for the first time that *in vitro *hyperglycemia and/or HIV-1 Nef enhance the lipid oxidation by releasing 8-iso-PGF2-alpha in astrocytes in addition to increased production of total nitrate. In this study, we observed that the production and release of 8-isoPF2-alpha was increased in glucose treated astrocytes in a dose-dependent manner as depicted in Figure 3A. The expression of Nef also increased more 8-isoPGF2-alpha in non-glucose treated control astrocytes. Various hyperglycemic conditions ranging from 10 to 20 mM glucose in combination with Nef significantly increased the production of 8 iso-PGF2-alpha in astrocytes released into medium. The *in vivo *results suggest a similar pattern, however the difference in iso-PGF-2-alpha production was higher between normal and diabetic mice brain. We also found that Nef expressed through HIV-1 based vectors or by SNV vectors showed a similar increase in the production of iso-PGF-2-alpha, indicating the exclusive effect of Nef protein on generating lipid oxidation reaction in CNS cells. Taken together, the results of the present study suggest the likely interactions between HIV-1 proteins and diabetes in inducing deleterious oxidative stress effects[6,9].

It has been reported that increased levels of ROS cause the loss of mitochondrial membrane permeability, which could induce alterations in F-actin dynamics [59]. Our results indicate that astrocytes under normal glycemic condition showed a dense cytoskeletal networking of F- actin in primary astrocytes, and variation in glycemic conditions caused a polarization of F-actin (figure 4A2) leading to disassembly (figure 4A2 and 4A3) in a dose-dependent manner. Similarly, exposure of astrocytes to various amounts of recombinant Nef protein resulted in depolarization of mitochondria in a dose-dependent manner, suggesting that the presence of extra Nef in astrocytes cause oxidation reaction in mitochondria, which may trigger caspase activity leading to apoptosis and cell death [34,38,60]. It has been reported that apoptotic-mediated stress-activation may occur by two distinct routes: one from the cell surface and the other from mitochondria as observed in this study (figure 4B2 and 4B3).

We also observed an upregulation in caspase-3 activity in a dose dependent fashion (figure 5B lanes 2 and 4) in astrocytes exposed to various glucose concentrations. The activation of caspase-3 was further enhanced by the addition of HIV-1 Nef [34]. The combination of hyperglycemia and Nef further activated the caspases in astrocytes (Figure 5B lane 6) as well as in diabetic mice, suggesting that Nef independently or in combination with hyperglycemia induces the apoptosis via caspases, which has been reported by our laboratory and other groups previously[14,38,60]. Interestingly, the expression of HIV-1 Nef alone was capable of activating caspase-3 in astrocytes. Similar observations have been reported by Lee *et al *(2005) in a study, demonstrating that Nef induced caspase-dependent apoptosis modulate the immune responses [60].

In conclusion, our study has demonstrated that diabetes and/or HIV-1 infection induce oxidative stress by enhancing the production of specific markers in human astrocytes and isolated brain tissues from diabetes-induced mice. Such up-regulation of pro-oxidative and pro-inflammatory pathways is a proof of concept that HIV-1 and hyperglycemic environment are able to induce extreme oxidative stress in HIV-1-infected individuals who are also diabetic. The results further suggest that hyperglycemic conditions and HIV-1 Nef, individually or in combination enhance apoptosis through the activation of procaspase-3, oxidation reaction species (ROS), lipid oxidation and complement factor C3, F-actin protein, mitochondrial depolarization as well as a decrease in the astrocytic cell marker protein GFAP. It is likely that individuals with hyperglycemia/diabetes may exhibit an accelerated progression of HIV-1 associated disorders including HAD. Finally, we are of the opinion that this study may provide new insights into the overall understanding of how hyperglycemia or diabetic conditions and HIV-1 protein Nef could interact with various cellular pathways in astrocytes.

## Competing interests

The authors declare that they have no competing interests.

## Authors' contributions

EAA carried out major molecular biology work including Western blots for caspases, HIV-1 Nef, GFAP (astrocytes marker), ELISAs and analysis of in vitro and in vivo data. CR generated the preliminary data for in vitro study including F-actin and mitochondrial staining in astrocytes. MM helped coordinating the study. AS contributed in manuscript and his suggestions were crucial for the study. MR participated in *in vivo *part of the study. His efforts include induction of diabetes in pups, viral injection in brain and the housing of mice. RP critically reviewed the study and gave his input. ZP designed and executed the study. She prepared HIV-1 and SNV based viral particles for *in vitro *and *in vivo *work, participated in viral injection in mice pups brain, supervised the entire study and drafted the manuscript. All authors have read and approved the final manuscript.

## References

[B1] Stein JH Klein MA Bellehumeur JL McBride PE Wiebe DA Otvos JD Sosman JM Use of human immunodeficiency virus-1 protease inhibitors is associated with atherogenic lipoprotein changes and endothelial dysfunction Circulation 2001 104 257 262 11457741 10.1161/01.cir.104.3.257

[B2] Kilby JM Tabereaux PB Severe hyperglycemia in an HIV clinic: preexisting versus drug-associated diabetes mellitus J Acquir Immune Defic Syndr Hum Retrovirol 1998 17 46 50 9436758 10.1097/00042560-199801010-00007

[B3] Monier PL Wilcox R Metabolic complications associated with the use of highly active antiretroviral therapy in HIV-1-infected adults Am J Med Sci 2004 328 48 56 15254441 10.1097/00000441-200407000-00007

[B4] Behrens G Dejam A Schmidt H Balks HJ Brabant G Korner T Stoll M Schmidt RE Impaired glucose tolerance, beta cell function and lipid metabolism in HIV patients under treatment with protease inhibitors Aids 1999 13 F63 70 10416516 10.1097/00002030-199907090-00001

[B5] Schurr A Rigor BM Brain anaerobic lactate production: a suicide note or a survival kit? Dev Neurosci 1998 20 348 357 9778571 10.1159/000017330

[B6] Mooradian AD Pathophysiology of central nervous system complications in diabetes mellitus Clin Neurosci 1997 4 322 326 9358975

[B7] Kolev OI Milanov I Central nervous system impairment in diabetic patients Electromyogr Clin Neurophysiol 1999 39 479 484 10627933

[B8] Mooradian AD Central nervous system complications of diabetes mellitus--a perspective from the blood-brain barrier Brain Res Brain Res Rev 1997 23 210 218 9164671 10.1016/s0165-0173(97)00003-9

[B9] Masliah E Ge N Mucke L Pathogenesis of HIV-1 associated neurodegeneration Crit Rev Neurobiol 1996 10 57 67 8853954 10.1615/critrevneurobiol.v10.i1.30

[B10] Sima AA Kamiya H Li ZG Insulin, C-peptide, hyperglycemia, and central nervous system complications in diabetes Eur J Pharmacol 2004 490 187 197 15094085 10.1016/j.ejphar.2004.02.056

[B11] Gao Q Gao YM Hyperglycemic condition disturbs the proliferation and cell death of neural progenitors in mouse embryonic spinal cord Int J Dev Neurosci 2007 25 349 357 17888615 10.1016/j.ijdevneu.2007.08.002

[B12] Aschner M Immune and inflammatory responses in the CNS: modulation by astrocytes Toxicol Lett 1998 102-103 283 287 10022267 10.1016/s0378-4274(98)00324-5

[B13] Stentz FB Kitabchi AE Hyperglycemia-induced activation of human T-lymphocytes with de novo emergence of insulin receptors and generation of reactive oxygen species Biochem Biophys Res Commun 2005 335 491 495 16084832 10.1016/j.bbrc.2005.07.109

[B14] Acheampong EA Parveen Z Muthoga LW Kalayeh M Mukhtar M Pomerantz RJ Human Immunodeficiency virus type 1 Nef potently induces apoptosis in primary human brain microvascular endothelial cells via the activation of caspases J Virol 2005 79 4257 4269 15767427 10.1128/JVI.79.7.4257-4269.2005 PMC1061575

[B15] Koedel U Kohleisen B Sporer B Lahrtz F Ovod V Fontana A Erfle V Pfister HW HIV type 1 Nef protein is a viral factor for leukocyte recruitment into the central nervous system J Immunol 1999 163 1237 1245 10415019

[B16] Cheng X Mukhtar M Acheampong EA Srinivasan A Rafi M Pomerantz RJ Parveen Z HIV-1 Vpr potently induces programmed cell death in the CNS in vivo DNA Cell Biol 2007 26 116 131 17328670 10.1089/dna.2006.0541

[B17] Renkema GH Saksela K Interactions of HIV-1 NEF with cellular signal transducing proteins Front Biosci 2000 5 D268 283 10704155 10.2741/renkema

[B18] Carl S Greenough TC Krumbiegel M Greenberg M Skowronski J Sullivan JL Kirchhoff F Modulation of different human immunodeficiency virus type 1 Nef functions during progression to AIDS J Virol 2001 75 3657 3665 11264355 10.1128/JVI.75.8.3657-3665.2001 PMC114857

[B19] Skowronski J Greenberg ME Lock M Mariani R Salghetti S Swigut T Iafrate AJ HIV and SIV Nef modulate signal transduction and protein sorting in T cells Cold Spring Harb Symp Quant Biol 1999 64 453 463 11232322 10.1101/sqb.1999.64.453

[B20] Bruder C Hagleitner M Darlington G Mohsenipour I Wurzner R Hollmuller I Stoiber H Lass-Florl C Dierich MP Speth C HIV-1 induces complement factor C3 synthesis in astrocytes and neurons by modulation of promoter activity Mol Immunol 2004 40 949 961 14725791 10.1016/j.molimm.2003.10.016

[B21] Speth C Stockl G Mohsenipour I Wurzner R Stoiber H Lass-Florl C Dierich MP Human immunodeficiency virus type 1 induces expression of complement factors in human astrocytes J Virol 2001 75 2604 2615 11222683 10.1128/JVI.75.6.2604-2615.2001 PMC115884

[B22] Cota M Kleinschmidt A Ceccherini-Silberstein F Aloisi F Mengozzi M Mantovani A Brack-Werner R Poli G Upregulated expression of interleukin-8, RANTES and chemokine receptors in human astrocytic cells infected with HIV-1 J Neurovirol 2000 6 75 83 10786999 10.3109/13550280009006384

[B23] Geleziunas R Xu W Takeda K Ichijo H Greene WC HIV-1 Nef inhibits ASK1-dependent death signalling providing a potential mechanism for protecting the infected host cell Nature 2001 410 834 838 11298454 10.1038/35071111

[B24] Kim S Shin JS Kim HJ Fisher RC Lee MJ Kim CW Streptozotocin-induced diabetes can be reversed by hepatic oval cell activation through hepatic transdifferentiation and pancreatic islet regeneration Lab Invest 2007 87 702 712 17483848 10.1038/labinvest.3700561

[B25] Naldini L Blomer U Gallay P Ory D Mulligan R Gage FH Verma IM Trono D In vivo gene delivery and stable transduction of nondividing cells by a lentiviral vector Science 1996 272 263 267 8602510 10.1126/science.272.5259.263

[B26] Abd-El-Basset EM Fedoroff S Upregulation of F-actin and alpha-actinin in reactive astrocytes J Neurosci Res 1997 49 608 616 9302082 10.1002/(SICI)1097-4547(19970901)49:5<608::AID-JNR11>3.0.CO;2-R

[B27] Parveen Z Mukhtar M Rafi M Wenger DA Siddiqui KM Siler CA Dietzschold B Pomerantz RJ Schnell MJ Dornburg R Cell-type-specific gene delivery into neuronal cells in vitro and in vivo Virology 2003 314 74 83 14517061 10.1016/s0042-6822(03)00402-1

[B28] Liu X Schrager JA Lange GD Marsh JW HIV Nef-mediated cellular phenotypes are differentially expressed as a function of intracellular Nef concentrations J Biol Chem 2001 276 32763 32770 11438519 10.1074/jbc.M101025200

[B29] Parveen Z Krupetsky A Engelstadter M Cichutek K Pomerantz RJ Dornburg R Spleen necrosis virus-derived C-type retroviral vectors for gene transfer to quiescent cells Nat Biotechnol 2000 18 623 629 10835599 10.1038/76458

[B30] Acheampong E Mukhtar M Parveen Z Ngoubilly N Ahmad N Patel C Pomerantz RJ Ethanol strongly potentiates apoptosis induced by HIV-1 proteins in primary human brain microvascular endothelial cells Virology 2002 304 222 234 12504564 10.1006/viro.2002.1666

[B31] Tawfik A Jin L Banes-Berceli AK Caldwell RB Ogbi S Shirley A Barber D Catravas JD Stern DM Fulton D Hyperglycemia and reactive oxygen species mediate apoptosis in aortic endothelial cells through Janus kinase 2 Vascul Pharmacol 2005 43 320 326 16257269 10.1016/j.vph.2005.08.018

[B32] Aronson D Hyperglycemia and the pathobiology of diabetic complications Adv Cardiol 2008 45 1 16 18230953 10.1159/000115118

[B33] Jacob BA Porter KM Elms SC Cheng PY Jones DP Sutliff RL HIV-1-induced pulmonary oxidative and nitrosative stress: exacerbated response to endotoxin administration in HIV-1 transgenic mouse model Am J Physiol Lung Cell Mol Physiol 2006 291 L811 819 16728526 10.1152/ajplung.00468.2005

[B34] Rasola A Gramaglia D Boccaccio C Comoglio PM Apoptosis enhancement by the HIV-1 Nef protein J Immunol 2001 166 81 88 11123279 10.4049/jimmunol.166.1.81

[B35] Stumbo AC Cortez E Rodrigues CA Henriques MG Porto LC Barbosa HS Carvalho L Mitochondrial localization of non-histone protein HMGB1 during human endothelial cell-Toxoplasma gondii infection Cell Biol Int 2008 32 235 238 17936030 10.1016/j.cellbi.2007.08.031

[B36] Strasberg P Bridge P Merante F Yeger H Pereira J Normal mitochondrial DNA and respiratory chain activity in familial dysautonomia fibroblasts Biochem Mol Med 1996 59 20 27 8902189 10.1006/bmme.1996.0059

[B37] Shi Y A structural view of mitochondria-mediated apoptosis Nat Struct Biol 2001 8 394 401 11323712 10.1038/87548

[B38] Saito Y Sharer LR Epstein LG Michaels J Mintz M Louder M Golding K Cvetkovich TA Blumberg BM Overexpression of nef as a marker for restricted HIV-1 infection of astrocytes in postmortem pediatric central nervous tissues Neurology 1994 44 474 481 8145918 10.1212/wnl.44.3_part_1.474

[B39] Schambelan M Benson CA Carr A Currier JS Dube MP Gerber JG Grinspoon SK Grunfeld C Kotler DP Mulligan K Management of metabolic complications associated with antiretroviral therapy for HIV-1 infection: recommendations of an International AIDS Society-USA panel J Acquir Immune Defic Syndr 2002 31 257 275 12439201 10.1097/00126334-200211010-00001

[B40] Mondal D Pradhan L Ali M Agrawal KC HAART drugs induce oxidative stress in human endothelial cells and increase endothelial recruitment of mononuclear cells: exacerbation by inflammatory cytokines and amelioration by antioxidants Cardiovasc Toxicol 2004 4 287 302 15470276 10.1385/ct:4:3:287

[B41] Gomez-Vera J de Alarcon A Jimenez-Mejias ME Acosta D Prados D Viciana P Hyperglycemia associated with protease inhibitors in HIV-1-infected patients Clin Microbiol Infect 2000 6 391 394 11168157 10.1046/j.1469-0691.2000.00083.x

[B42] Marfella R Quagliaro L Nappo F Ceriello A Giugliano D Acute hyperglycemia induces an oxidative stress in healthy subjects J Clin Invest 2001 108 635 636 11518739 10.1172/JCI13727 PMC209408

[B43] Fischer M Joos B Hirschel B Bleiber G Weber R Gunthard HF Cellular viral rebound after cessation of potent antiretroviral therapy predicted by levels of multiply spliced HIV-1 RNA encoding nef J Infect Dis 2004 190 1979 1988 15529263 10.1086/425983

[B44] Schrager JA Marsh JW HIV-1 Nef increases T cell activation in a stimulus-dependent manner Proc Natl Acad Sci USA 1999 96 8167 8172 10393966 10.1073/pnas.96.14.8167 PMC22206

[B45] Guerri C Renau-Piqueras J Alcohol, astroglia, and brain development Mol Neurobiol 1997 15 65 81 9396005 10.1007/BF02740616

[B46] Fiala M Rhodes RH Shapshak P Nagano I Martinez-Maza O Diagne A Baldwin G Graves M Regulation of HIV-1 infection in astrocytes: expression of Nef, TNF-alpha and IL-6 is enhanced in coculture of astrocytes with macrophages J Neurovirol 1996 2 158 166 8799208 10.3109/13550289609146878

[B47] Hockett PK Emery SC Hansen L Masliah E Evidence of oxidative stress in the brains of fetuses with CNS anomalies and islet cell hyperplasia Pediatr Dev Pathol 2004 7 370 379 15383932 10.1007/s10024-001-0130-2

[B48] Sacktor N Haughey N Cutler R Tamara A Turchan J Pardo C Vargas D Nath A Novel markers of oxidative stress in actively progressive HIV dementia J Neuroimmunol 2004 157 176 184 15579295 10.1016/j.jneuroim.2004.08.037

[B49] Turchan J Pocernich CB Gairola C Chauhan A Schifitto G Butterfield DA Buch S Narayan O Sinai A Geiger J Oxidative stress in HIV demented patients and protection ex vivo with novel antioxidants Neurology 2003 60 307 314 12552050 10.1212/01.wnl.0000042048.85204.3d

[B50] Otis JS Ashikhmin YI Brown LA Guidot DM Effect of HIV-1-related protein expression on cardiac and skeletal muscles from transgenic rats AIDS Res Ther 2008 5 8 18439274 10.1186/1742-6405-5-8 PMC2365956

[B51] Price TO Ercal N Nakaoke R Banks WA HIV-1 viral proteins gp120 and Tat induce oxidative stress in brain endothelial cells Brain Res 2005 1045 57 63 15910762 10.1016/j.brainres.2005.03.031

[B52] Brunner T Mogil RJ LaFace D Yoo NJ Mahboubi A Echeverri F Martin SJ Force WR Lynch DH Ware CF Cell-autonomous Fas (CD95)/Fas-ligand interaction mediates activation-induced apoptosis in T-cell hybridomas Nature 1995 373 441 444 7530336 10.1038/373441a0

[B53] Dobmeyer TS Findhammer S Dobmeyer JM Klein SA Raffel B Hoelzer D Helm EB Kabelitz D Rossol R Ex vivo induction of apoptosis in lymphocytes is mediated by oxidative stress: role for lymphocyte loss in HIV infection Free Radic Biol Med 1997 22 775 785 9119245 10.1016/s0891-5849(96)00403-0

[B54] Fiebich BL Hull M Lieb K Gyufko K Berger M Bauer J Prostaglandin E2 induces interleukin-6 synthesis in human astrocytoma cells J Neurochem 1997 68 704 709 9003059 10.1046/j.1471-4159.1997.68020704.x

[B55] Grillo CA Piroli GG Rosell DR Hoskin EK McEwen BS Reagan LP Region specific increases in oxidative stress and superoxide dismutase in the hippocampus of diabetic rats subjected to stress Neuroscience 2003 121 133 140 12946706 10.1016/s0306-4522(03)00343-9

[B56] Montine TJ Sidell KR Crews BC Markesbery WR Marnett LJ Roberts LJ Morrow JD Elevated CSF prostaglandin E2 levels in patients with probable AD Neurology 1999 53 1495 1498 10534257 10.1212/wnl.53.7.1495

[B57] Bukrinsky MI Nottet HS Schmidtmayerova H Dubrovsky L Flanagan CR Mullins ME Lipton SA Gendelman HE Regulation of nitric oxide synthase activity in human immunodeficiency virus type 1 (HIV-1)-infected monocytes: implications for HIV-associated neurological disease J Exp Med 1995 181 735 745 7530762 10.1084/jem.181.2.735 PMC2191885

[B58] Akaike T Role of free radicals in viral pathogenesis and mutation Rev Med Virol 2001 11 87 101 11262528 10.1002/rmv.303 PMC7169086

[B59] Gourlay CW Ayscough KR A role for actin in aging and apoptosis Biochem Soc Trans 2005 33 1260 1264 16246093 10.1042/BST0331260

[B60] Lee SB Park J Jung JU Chung J Nef induces apoptosis by activating JNK signaling pathway and inhibits NF-kappaB-dependent immune responses in Drosophila J Cell Sci 2005 118 1851 1859 15827086 10.1242/jcs.02312

